# Analysis of the genetic basis of plant height-related traits in response to ethylene by QTL mapping in maize (*Zea mays* L.)

**DOI:** 10.1371/journal.pone.0193072

**Published:** 2018-02-21

**Authors:** Weiqiang Zhang, Zhi Li, Hui Fang, Mingcai Zhang, Liusheng Duan

**Affiliations:** 1 State Key Laboratory of Plant Physiology and Biochemistry, Engineering Research Center of Plant Growth Regulator, Ministry of Education, College of Agronomy and Biotechnology, China Agricultural University, Haidian District, Beijing, China; 2 National Maize Improvement Center of China, College of Agronomy and Biotechnology, China Agricultural University, Haidian District, Beijing, China; Western Australia Department of Agriculture and Food, AUSTRALIA

## Abstract

Ethylene (ET) is critical importance in the growth, development, and stress responses of plants. Plant hormonal stress responses have been extensively studied, however, the role of ET in plant growth, especially plant height (PH) remains unclear. Understanding the genetic control for PH in response to ET will provide insights into the regulation of maize development. To clarify the genetic basis of PH-related traits of maize in response to ET, we mapped QTLs for PH, ear height (EH), and internode length above the uppermost ear (ILAU) in two recombinant inbred line (RIL) populations of *Zea mays* after ET treatment and in an untreated control (CK) group. Sixty QTLs for the three traits were identified. Twenty-two QTLs were simultaneously detected under both ET treatment and untreated control, and five QTLs were detected at two geographic locations under ET treatment only. Individual QTL can be explained 3.87–17.71% of the phenotypic variance. One QTL (*q2PH9-1*, *q1PH9*, *q1EH9*/*q1ILAU9-1*, *q2ILAU9*, and *q2EH9*) for the measured traits (PH, EH, ILAU) was consistent across both populations. Two QTLs (*q2PH2-5*, *q2ILAU2-2*, *q1PH2-2*, and *q1ILAU2-2*; *q1PH8-1*, *q1EH8-1*, *q2PH8-1*) were identified for up to two traits in both locations and populations under both ET treatment and untreated control. These consistent and stable regions are important QTLs of potential hot spots for PH, ear height (EH), and internode length above the uppermost ear (ILAU) response to ET in maize; therefore, QTL fine-mapping and putative candidate genes validation should enable the cloning of PH, EH, and ILAU related genes to ET response. These results will be valuable for further fine-mapping and quantitative trait nucleotides (QTNs) determination, and elucidate the underlying molecular mechanisms of ET responses in maize.

## Introduction

The gaseous endogenous plant hormone ethylene (ET) is important for plant growth and development [1–3]. By restricting cell elongation and regulating cell division, ET is most commonly associated with cell size regulation [4, 5]. In terms of development, ET is thought to be an ‘aging’ hormone, due to the role it plays in accelerating such processes as abscission, senescence, and ripening [6–8]. Components of the ET signal transduction pathway in *Arabidopsis* have been identified through genetic approaches. The basic model of ET signal transduction works as follows: ET receptors (ETR1, ERS1, ETR2, ERS2 and EIN4) and receptors activating CTR1 in the absence of ET keeps downstream signaling components EIN2 and EIN3 inactive. Upon binding ET, the receptors no longer activate CTR1, while EIN2 activates the EIN3/EIL transcription factors, thus inducing a transcriptional cascade and the establishment of ET responses [3, 9, 10].

ET normally causes the inhibition of stem elongation [6, 11]. However, ET treatment also causes a stunted and thick inflorescence stem, which is also observed in the untreated *ctr1* mutant of *Arabidopsis* [12]. Rapid shoot growth in aquatic species is controlled by the levels of ET synthesis and action. Thus, the elongation response of deepwater rice, commonly known as ‘supergrowth’, is primarily dependent on the hypoxic induction of ACC synthase [12–14]. The resulting increase in ET modulates the balance between gibberellic acid (GA) and abscisic acid (ABA) and induces stem elongation [12, 15–16]. Plant height has been shown to decrease with decreasing internode length upon ET application in maize, barley, oats, and wheat [17–22]. In *Rumex* species, ET sensitivity was shown to be the key factor controlling submergence-induced shoot elongation [23]. These observations confirm that ET is indispensable for internode elongation in higher plants. Therefore, further studies ET-responsive gene of stem elongation related traits (e.g., PH) is important for reveal the molecular mechanisms of ET signal transduction cascade and interacting with other plant hormones. Multiple QTLs for PH and ear height have been detected using different populations in maize [24–28], and genetic analysis of ear to plant heights (EPR, ear height /plant height) in relation to ET also was reported lately [29]. However, these results do not provide enough data to clarify maize genomic regions of PH-related traits response to ET.

In the present study, to explore the genetic architecture for PH-related (PH, ear height, and internode length above the uppermost ear) traits response to ET in maize plant, QTL mapping of these traits (with ET treatment or without) was conducted using two recombinant inbred line (RIL) populations derived from the cross K22 × BY815 and KUI3 × B77. The objectives of this study were: (1) to determine QTLs of additional genome regions of traits relating to PH, ear height (EH), and internode length above the uppermost ear (ILAU) under ET treatment conditions; (2) to estimate their differences in QTLs detected under both ET treatment and untreated control; and (3) to characterize and analyze the QTLs and candidate gene and compare the differences between the two RILs population associated with ET response.

## Materials and methods

### Plant materials and field experiments

The two sets of RIL populations for this research, Pop. 1 (N = 197 RILs) and Pop. 2 (N = 177 RILs), were derived from the cross K22 × BY815 and KUI3 × B77, and were developed by China Agricultural University in a single-seed descent method. Parental inbred lines BY815, K22, and B77 were derived from a Chinese Non-Stiff Stalk germplasm, whereas the inbred line KUI3 was derived from CIMMYT and tropical germplasm. By815 and B77 is sensitive to ET treatment, while not K22 and KU13.

A field experiment was conducted during the growing seasons in 2015, the two populations and four parents were evaluated under two treatments, with and without ethylene administration, and in two locations, the Wuqiao test station (Hebei Province, WQ) and the Lishu test station (Jilin Province, LS). No specific permissions were required in the two experimental site. The field studies did not involve wildlife or any endangered or protected species. All the maize inbred lines of flat planting were hand-sown on April 26 in 2015 at Wuqiao (WQ), and ridge planting on May 6 in 2015 at Lishu (LS), respectively. A split–split–plot design was used in the field experiments with two replications. The main plot was ET treatment or control (CK) (with two levels), the sub-plot factor was populations (with two levels), and the sub-sub-plot factor was genotype [24, 30]. Each plot consisted of a row of 3m × 0.67m with a density of 67,500 plants/ha. For ET treatment, ethephon (270 g/ha at a 600 mg/L concentration, as used by Wei [31]) was applied by foliar-spraying with an agricultural manual sprayer at the 8-leaf stage (V8, according to Abendroth et al.) [32, 33]. In the control group, an equal volume of water was applied by foliar-spraying at the same stage of growth. Treatment was performed quarantine by a membrane or baffle and applied on June 16 (2015) at Wuqiao (WQ) and on June 22 (2015) at Lishu (LS), respectively.

Twenty days after pollen shedding, eight consecutive plants from the plot center were selected to evaluate PH, EH, and ILAU. PH was measured from ground to tassel top, EH from ground to ear node, and ILAU from the node above the uppermost ear to tassel top. The eight-plant average in each replication is reported as the trait values per family, while the average under the two experimental environments in the treatment conditions is described as the overall performance. Broad-sense heritability (*h*^*2*^) was calculated as follows: *h*^*2*^ = *σ*_*g*_^*2*^/ (*σ*_*g*_^*2*^ + *σ*_*ge*_^*2*^*/n* + *σ*_*e*_^*2*^*/nr*), where *σ*_*g*_^*2*^ is the genetic variance, *σ*_*ge*_^*2*^ is interaction variance of genotype and environment, *σ*_*e*_^*2*^ is error variance, *n* represents the number of environments, *r* is replication number. Estimation of *σ*_*g*_^*2*^, *σ*_*ge*_^*2*^, and *σ*_*e*_^*2*^, and descriptive statistics and simple Pearson correlation coefficients (r), and analysis of variance (ANOVA) were performed using SPSS 21.0 [34–36].

### Molecular linkage map construction and QTL analysis

Genomic DNA was obtained from leaves of seedling stage plants from the two RIL populations using the cetyltrimethylammonium bromide method (CTAB) [37]. Genotype analysis of each SNP marker was conducted with the Illumina MaizeSNP50 BeadChip and analyzed using Genome Studio Data analysis software (Illumina Inc., San Diego, CA, USA), generating clusters of homozygous and heterozygous genotypes. In total, 3072 SNP markers were selected to analyze polymorphisms between KUI3×B77 and K22×BY815. A total of 2126 and 2263 SNP markers had polymorphisms between parent pairs [38, 39]. After excluding SNP markers with major segregation distortion, 2126 and 2263 SNP markers were used to generate two genetic linkage maps by Joinmap 4.0 software [34]. The maps were 1744 cM in length (average mapping interval of 0.82 cM) for Pop. 1 and 1640.4 cM (average mapping interval of 0.74 cM) for Pop. 2. A total of 4136 SNP loci were consistent with maize database chromosome bin locations.

QTL mapping for each location was conducted by composite-interval mapping (CIM) in Windows QTL cartographer version 2.5 [40, 41]. For CIM, Model 6 of Zmaoqtl module was applied for detecting QTL and their effects. The genome was scanned every 1 cM between markers and putative QTLs with a window size of 10 cM. Maximum cofactors were utilized to manipulate trait genetic backgrounds. Five control markers were determined by forward and backward regression. Empirical threshold levels for declaring QTL significance at the 5% genome-wide type I error level were achieved via 1000 random permutations [34, 42]. Estimates of phenotypic variance and effect were based on expressed values of QTL peak.

## Results

### Phenotypic variance in plant height, ear height, and internode length above the uppermost ear under ethylene treatment

According to the combined ANOVA analysis across the two locations, all components were highly significant variance in all measured traits of RIL populations, except for replication variance (Table 1). PH-related trait values for the parents and RIL population in the two treatment groups in Table 2. PH, EH, and ILAU for parent KUI3 were less than for B77. The trait values of the population demonstrated high variance, showing continuous distribution around average and transgressive segregations that exceeded high or low parent values (Table 2). ET treatment was effective (*P* < 0.01) and led to a decrease in PH, EH, and ILAU (except ILAU of KUI3). Using the population KUI3×B**77** and parent B77 as examples, the average effect of ET treatment on the population was a decrease in PH from 170.16 to 150.52 cm, EH from 71.53 to 54.95 cm, and ILAU from 98.59 to 95.71 cm (*P* < 0.01). The average effect of ET treatment on B77 was a decrease in PH from 191.33 to 160.29 cm, EH from 73.18 to 53.23 cm, and ILAU from 116.60 to 107.06 cm (*P* < 0.01). The broad-sense heritability for all traits in the ET treated and control groups was from 0.45 to 0.84 (Table 2).

**Table 1:** Combined analysis of variance for plant height-related traits in the ethylene treated and control RIL populations grown in two different locations (*F*-values).

Variation sources	KUI3×B77		
	PH^a^	EH^b^	ILAU^c^
Family	9.87**	8.33**	7.99**
Location	3.96**	4.12**	3.22**
Replication	1.43	1.33	0.95
Treatment	556.77**	229.81**	29.39**
Family×location	1.30	1.27	0.77
Family×treatment	2.67**	2.73**	3.58**

^a^ *PH* plant height.

^b^ *EH* ear height.

^c^ *ILAU* the internode length above the uppermost ear.

** Significant at *P* < 0.01.

**Table 2:** Phenotypic performance of plant height-related traits for two RIL populations and their parents under two ethylene treatments.

Population		PH^a^		EH^b^		ILAU^c^	
		CK^d^	ET^e^	CK^d^	ET^e^	CK^d^	ET^e^
B77	Mean±SD	191.33±4.70	160.29±6.26	73.18±2.22	53.23±2.95	116.60±3.83	107.06±4.78
KUI3	Mean±SD	157.50±4.67	145.50±5.72	68.00±3.12	56.91±2.76	89.50±2.54	90.11±3.87
KUI3×B77	Mean±SD	170.16±1.03	150.52±0.94	71.53±0.66	54.95±0.47	98.59±0.65	95.71±0.58
	Range	135.24–212.87	116.49–182.4	51.8–90.76	40.57–71.81	77.68–124.79	76.88–120.89
	Kurtosis	-0.07	-0.11	-0.53	-0.12	0.04	0.20
	Skewness	-0.21	-0.30	-0.01	0.08	0.06	0.02
	H_B_^2^	0.75	0.71	0.80	0.73	0.65	0.57
	CI	0.67–0.80	0.62–0.77	0.74–0.84	0.65–0.79	0.55–0.73	0.45–0.67

^a^ *PH* plant height.

^b^ *EH* ear height.

^c^ *ILAU* the internode length above the uppermost ear.

^d^ *CK* without ethylene (CK) treatments.

^e^ *ET* with ethylene (ET) treatments.

*h*^2^ the broad-sense heritability.

*CI* Confidence interval, the confidence intervals of broad-sense heritability between 5 and 95% significance levels.

The traits showed phenotypic correlations under the treatment conditions (Table 3). In all treatment conditions, pairwise correlations were significant with the exception of CK_ILAU, CK_EH, and ET_EH.

**Table 3:** Correlation coefficients between plant height-related traits in the two RIL populations.

Trait	CK_PH ^a^	ET_PH ^b^	CK_EH ^c^	ET_EH ^d^	CK_ILAU ^e^	ET_ILAU ^f^
CK_PH ^a^						
ET_PH ^b^	0.85**					
CK_EH ^c^	0.67**	0.62**				
ET_EH ^d^	0.53**	0.68**	0.83**			
CK_ILAU ^e^	0.80**	0.65**	0.10	0.04		
ET_ILAU ^f^	0.78**	0.87**	0.27**	0.23**	0.83**	

Correlation coefficients below the diagonal line in each quadrant of the table are for the KUI3×B77 population.

^a^ *CK_PH* plant height without ethylene (CK) treatments.

^b^ *ET_PH* plant height with ethylene (ET) treatments.

^c^ *CK_EH* ear height without ethylene (CK) treatments.

^d^ *ET_EH* ear height with ethylene (ET) treatments.

^e^ *CK_ILAU* the internode length above the uppermost ear without ethylene (CK) treatments.

^f^ *ET_ILAU* the internode length above the uppermost ear with ethylene (ET) treatments.

** Significant at *P* < 0.01.

### QTL identification for the objective agronomic traits

**Fig 1:**
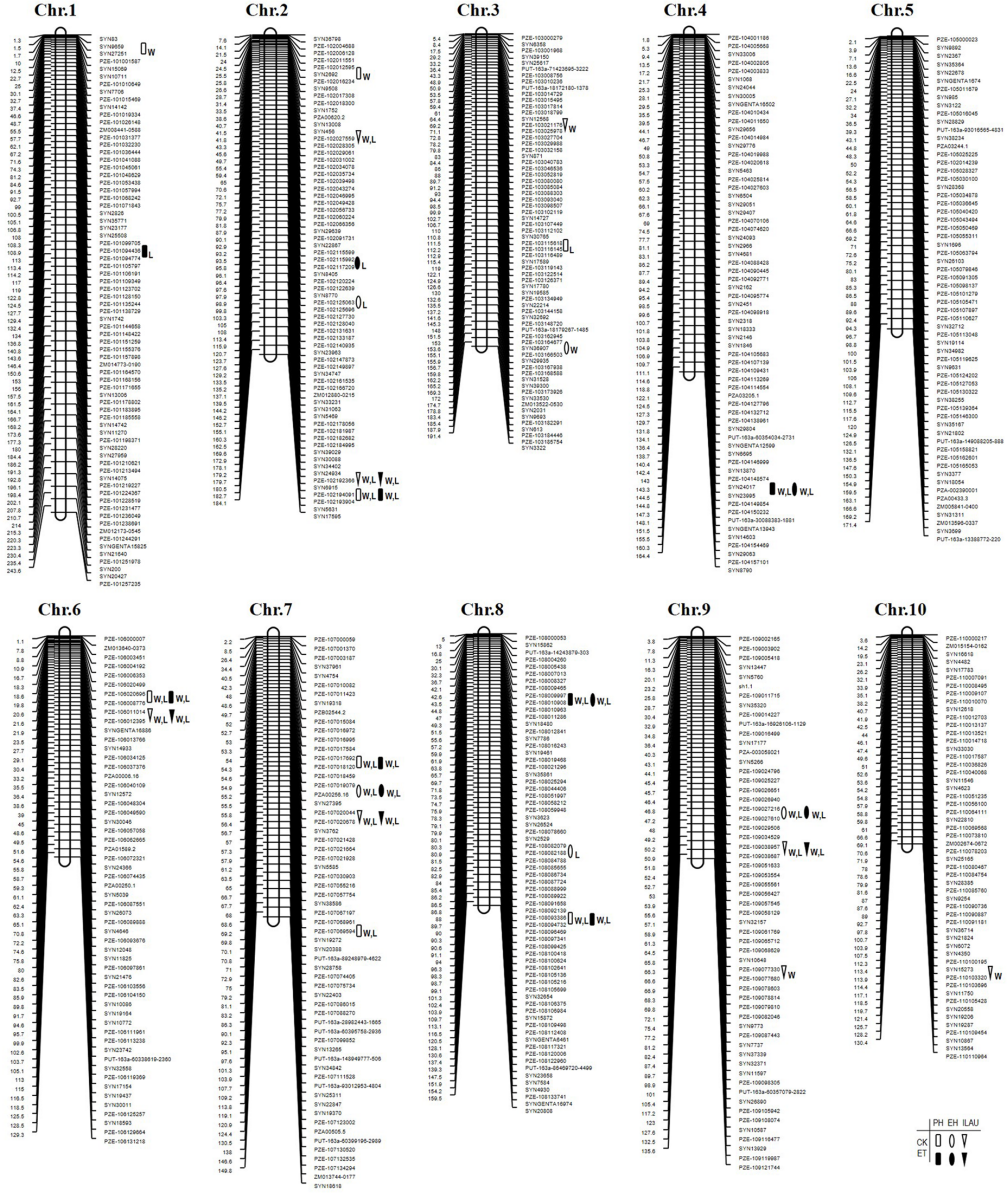
Chromosomal locations of QTLs for plant height-related traits in Pop. 1 (K22 × BY815, N = 197 RILs) population across two environments and two ethylene treatments. Note: Rectangle QTL detected for plant height (PH) with ethylene treatment (Solid) or without (Hollow), Oval QTL detected for ear height (EH) with ethylene treatment (Solid) or without (Hollow), and Inverted triangle QTL detected for internode length above the uppermost ear (ILAU) with ethylene treatment (Solid) or without (Hollow). W indicates a QTL detected in Wuqiao test station, and L indicates a QTL detected in Lishu test station.

**Fig 2:**
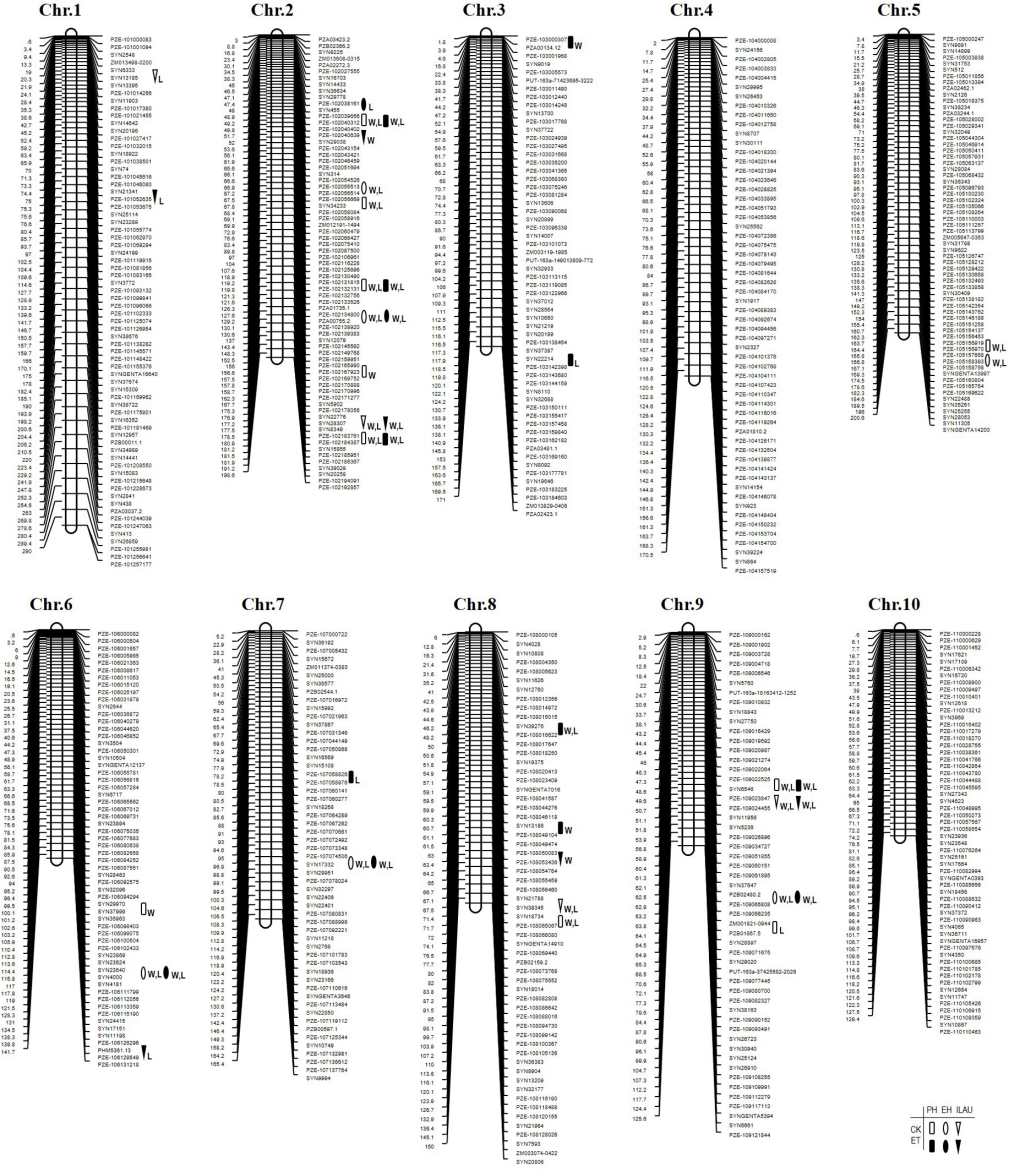
Chromosomal locations of QTLs for plant height-related traits in Pop. 2 (KUI3 × B77, N = 177 RILs) population across two environments and two ethylene treatments. Note: Rectangle QTL detected for plant height (PH) with ethylene treatment (Solid) or without (Hollow), Oval QTL detected for ear height (EH) with ethylene treatment (Solid) or without (Hollow), and Inverted triangle QTL detected for internode length above the uppermost ear (ILAU) with ethylene treatment (Solid) or without (Hollow). W indicates a QTL detected in Wuqiao test station, and L indicates a QTL detected in Lishu test station.

Sixty QTLs related to the three traits were identified in both populations (Table 4) by CIM, locating 30 QTLs in Pop. 1 (Fig 1), and 30 QTLs in Pop. 2 (Fig 2). Twenty-two QTLs were simultaneously detected in ET-treated and control groups, while five QTLs were detected at two locations under ET treatment only. Individual QTL explained 3.87 to 17.71% of phenotypic variance.

**Table 4:** QTLs detected for plant height-related traits in the two RIL populations across two environments and two ethylene treatments.

Traits	QTL	Chr.	Location ^a^	Treatment ^b^	Position ^c^ (cM)	Marker Interval ^d^	Support interval ^e^ (cM)	Physical interval ^f^ (bp)	LOD ^g^	R^2^ (%) ^h^	A^i^	Positive allele ^j^
KUI3 × B77												
PH	*q2PH2-1*	2	LS	CK	49.2	PZE-102038161- SYN29038	47.1–51.7	18,316,951–20,724,795	3.47	6.45	-3.48	B77
			LS	ET					8.23	14.91	-5.06	
			WQ	CK					4.26	7.96	-4.23	
			WQ	ET					4.01	7.38	-3.99	
	*q2PH2-2*	2	WQ	CK	68.7	PZE-102056514- ZM012191-1494	66.9–69.1	34,539,814–37,198,403	5.92	11.31	-6.43	B77
			LS	CK					3.68	6.97	-3.69	
	*q2PH2-3*	2	WQ	CK	118.9	PZE-102132131- PZE-102134800	119.2–126.3	182,620,244–184,968,231	4.23	8.12	5.41	KUI3
			WQ	ET					3.62	6.97	3.85	
			LS	CK					5.24	9.25	5.63	
			LS	ET					3.26	5.84	2.97	
	*q2PH2-4*	2	WQ	CK	157.2	PZE-102169752- PZE-102170996	156.6–157.8	213,205,091–214,210,573	2.59	4.8	-4.05	B77
	*q2PH2-5*	2	LS	CK	180.9	PZE-102184387- SYN15855	178.5–180.9	227,531,180–228,895,610	2.63	4.81	-3	B77
			LS	ET					2.89	5.12	-2.68	
			WQ	CK					3.12	5.01	-4.16	
			WQ	ET					2.67	6.23	-3.37	
	*q2PH3*	3	LS	ET	118.2	SYN37387- PZE-103144159	116.5–119.8	195,935,474–199,484,261	3.9	6.74	3.41	KUI3
	*q2PH5*	5	WQ	CK	164.4	PZE-105156919- PZE-105158393	162.3–165.8	205,567,299–206,188,028	3.66	6.93	4.77	KUI3
			LS	CK					4.02	7.71	3.95	
	*q2PH6*	6	WQ	CK	97.2	PZE-106094294- PZE-106098403	95.2–101.2	149,598,681–152,167,281	5.37	10.31	-5.67	B77
	*q2PH7*	7	LS	ET	78.5	PZE-107058976- PZE-107060141	78.2–78.5	112,980,785–115,691,953	2.95	4.6	2.84	KUI3
	*q2PH8-1*	8	LS	ET	45.8	PZE-108015015- PZE-108018250	43.9–50	14,753,943–17,383,010	6.67	12.15	-4.52	B77
			WQ	ET					5.36	11.47	-3.37	
	*q2PH8-2*	8	WQ	ET	63	PZE-108046118- PZE-108054764	59.9–63.4	76,039,534–97,777,183	5	8.98	-4.24	B77
	*q2PH8-3*	8	LS	CK	71.7	PZE-108064061- SYNGENTA14910	70.8–72	114,297,738–118,401,344	7.22	14.47	-5.2	B77
			WQ	CK					6.35	11.51	-6.32	
	*q2PH9-1*	9	LS	ET	48.3	PZE-109022525- PZE-109024455	46.3–49.6	23,008,509–24,487,068	6.12	10.77	4.33	KUI3
			WQ	ET					4.21	7.06	3.84	
			LS	CK					6.38	12.14	4.2	
			WQ	CK					5.41	10.12	5.32	
	*q2PH9-2*	9	LS	CK	62.9	PZB02480.2- PZE-109071675	62.1–64.5	107,416,972–116,353,876	3.97	7.56	3.77	KUI3
EH	*q2EH2-1*	2	LS	ET	47.4	SYN29778- SYN29038	46.5–51.7	17,793,282–20,724,795	4.76	8.69	-2.47	B77
	*q2EH2-2*	2	WQ	CK	66.9	PZE-102054526- SYN34233	66.1–67.5	32,516,815–36,590,474	5.08	9.12	-3.22	B77
			LS	CK					3.04	5.81	-2.47	
	*q2EH2-3*	2	WQ	CK	129.2	PZE-102134800- PZE-102139383	126.3–130.1	184,968,231–187,633,354	3.08	5.75	1.51	KUI3
			WQ	ET					2.89	5.07	1.72	
			LS	CK					3.56	6.24	2.97	
			LS	ET					3.25	5.33	2.86	
	*q2EH3*	3	WQ	ET	1.8	PZE-103000307- PZE-103001968	1.5–3.9	1,233,964–1,978,736	2.52	4.55	-1.35	B77
	*q2EH5*	5	WQ	CK	164.4	PZE-105156970- PZE-105158756	163.7–166.8	205,776,348–206,557,530	5.45	9.98	2.93	KUI3
			LS	CK					3.69	7.18	2.8	
	*q2EH6*	6	LS	CK	116.1	SYN23640- PZE-106112056	113.6–117.8	156,879,515–159,464,206	3.95	7.6	-2.8	B77
			WQ	CK					4.81	8.56	-2.77	
			WQ	ET					9.18	17.71	-2.67	
			LS	ET					2.74	4.47	-1.72	
	*q2EH7*	7	LS	ET	96.1	PZE-107074506- SYN32297	94.6–99.1	130,360,641–133,374,241	5.64	10.16	2.63	KUI3
			WQ	ET					2.64	4.46	1.37	
			LS	CK					3.11	5.93	2.52	
			WQ	CK					5.32	9.54	2.41	
	*q2EH9*	9	LS	CK	62.5	PZE-109065808- PZE-109066235	62.5–62.9	108,522,923–109,304,376	3.28	6.28	2.6	KUI3
			WQ	ET					3.51	6.41	1.62	
			LS	ET					5.1	9.18	2.55	
			WQ	CK					4.32	7.54	2.01	
ILAU	*q2ILAU1-1*	1	LS	ET	19	SYN13185- SYN13395	19.0–20.3	6,844,116–7,565,560	2.82	5.49	-2.56	B77
	*q2ILAU1-2*	1	LS	CK	74	PZE-101046080- SYN23289	71.3–75.6	31,850,441–38,871,055	5.06	10.28	-3.48	B77
	*q2ILAU2-1*	2	WQ	ET	49.8	PZE-102040312- PZE-102043154	48.9–52	20,103,068–21,874,416	4.08	7.54	-2.9	B77
	*q2ILAU2-2*	2	LS	CK	180.9	SYN28307- PZE-102186367	176.9–181.5	225,537,147–230,291,137	4.09	7.92	-3.01	B77
			LS	ET					4.5	9.06	-3.42	
			WQ	CK					4.09	7.92	-3.01	
			WQ	ET					4.5	9.06	-3.42	
	*q2ILAU6*	6	LS	ET	141.7	PHM5361.13- PZE-106129549	139.8–141.7	166,592,099–167,325,854	2.86	5.63	2.61	B77
	*q2ILAU8-1*	8	WQ	ET	63	PZE-108049474- PZE-108055459	61.1–64.2	85,149,829–99,749,632	6.05	11.34	-3.42	B77
	*q2ILAU8-2*	8	WQ	CK	69.5	SYN39345- PZE-108066080	67.1–71.7	106,271,779–117,871,384	4.65	9.74	-3.5	B77
			LS	CK					6.82	13.8	-3.99	
	*q2ILAU9*	9	WQ	ET	47.3	PZE-1090212740 SYN11956	45.4–50.7	21,678,811–25,817,148	4.8	10.87	2.9	KUI3
			LS	ET					4.77	8.67	3.22	
			WQ	CK					4.72	9.94	3.98	
			LS	CK					6.94	13.02	4.27	
K22 × BY815												
ILAU	*q1ILAU2-1*	2	WQ	CK	41.5	PZE-102027559- PZE-102028305	41.5–41.8	12,801,930–13,300,441	4.48	7.46	2.7	BY815
			LS	CK					2.98	4.92	2.29	
	*q1ILAU2-2*	2	WQ	CK	180.5	PZE-102192366- PZE-102193904	179.2–182.7	234,718,008–236,615,130	4.61	10.82	-2.73	K22
			LS	ET					5.37	8.71	-4.05	
			LS	CK					4.63	8.12	-2.95	
			WQ	ET					6.07	11.07	-3.03	
	*q1ILAU3*	3	WQ	CK	70.5	SYN12568- PZE-103027704	61–71.1	12,189,577–20,378,743	3.13	4.76	-2.1	K22
	*q1ILAU6*	6	LS	CK	21.9	PZE-106020696- PZE-106013766	18.3–28.8	16,795,395–34,863,238	4.58	7.7	3.75	BY815
			LS	ET					4.86	7.65	3.73	
			WQ	CK					4	6.58	2.48	
			WQ	ET					3.92	6.74	2.35	
	*q1ILAU7-1*	7	LS	ET	54.3	PZE-107015084- SYN27395	49.7–55.2	11,799,137–17,697,488	3.53	6.19	3.34	BY815
			LS	CK					3.85	6.65	3.73	
			WQ	ET					3.68	5.81	2.48	
			WQ	CK					3.23	5.7	2.35	
	*q1ILAU7-2*	7	WQ	CK	70.1	PZE-107068961- SYN28758	68–70.8	125,861,581–129,967,283	3.32	5.48	2.29	BY815
			LS	CK					4.06	6.9	2.75	
	*q1ILAU8*	8	WQ	CK	94.8	PZE-108099425- PZE-108105216	90.3–98.3	155,643,006–159,952,552	3.7	6.12	-2.47	K22
			LS	CK					4.35	7.35	-2.89	
			WQ	ET					2.77	4.73	-2.1	
			LS	ET					5.03	8.43	-3.62	
	*q1ILAU9-1*	9	LS	CK	50.9	PZE-109027610- PZE-109055561	46.8–52.4	28,005,182–89,325,234	3.6	6.41	2.55	BY815
			WQ	ET					3.59	6.26	2.29	
			LS	ET					5.57	9.04	4.03	
			WQ	CK					3.81	7.22	2.72	
	*q1ILAU9-3*	9	WQ	CK	66.3	PZE-109077680- SYN12671	66.3–69.4	125,169,744–128,964,637	2.53	3.87	1.91	BY815
	*q1ILAU10*	10	WQ	CK	113.4	PZE-110103320- PZE-110103696	113.4–113.9	146,173,534–146,292,761	2.67	4.08	-1.96	K22

*PH* plant height, *EH* ear height, *ILAU* the internode length above the uppermost ear.

^a^ *Location* QTL detected in Wuqiao (WQ) and Lishu (LS).

^b^ *Treatment CK*, without ethylene (CK) treatments; *ET*, with ethylene (ET) treatments.

^c^ *Position* The peak position with the highest LOD of each QTL.

^d^ *Marker Interval* Flanking markers, the left and right markers of the QTL.

^e^ *Support interval* Genetic position interval of each QTL.

^f^ *Physical interval* Physical location of the QTL in the maize genome (MaizeDB; http://www.maizegdb.org/).

^g^ *LOD scores* logarithm of the odds (to the base 10).

^h^ *R*^*2*^ *(%)* rate of contribution were calculated for each QTL.

^i^ *A* additive effect, positive values indicated that BY815 or KUI3 carries the allele for an increase in the traits, while negative values(-) indicated that K22 or B77 contributed the allele for an increase in the trait value.

^j^ *Positive allele* effect carries of parental.

### QTL analysis for plant height

Twenty-five QTLs were identified for PH under ET-treated and control groups in the two populations (Table 4), with 11 QTLs in Pop. 1 (Fig 1), and 14 QTLs in Pop. 2 (Fig 2) respectively. These QTLs were mapped onto all chromosomes except for chromosomes 5 and 10, and an individual QTL explained 4.45 to 14.91% of the phenotypic variance. Six of the 25 QTLs from KUI3 (*q2PH2-3*, *q2PH3*, *q2PH5*, *q2PH7*, *q2PH9-1*, *q2PH9-2*) caused PH values to rise. Four QTLs (*q2PH2-1*, *q2PH2-3*, *q2PH2-5*, *q2PH9-1*) were identified in both ET-treated and control conditions, while one QTL (*q2PH8-1*) were observed under ET treatment, and three (*q2PH2-2*, *q2PH5*, *q2PH8-3*) under control conditions. Indeed, QTL *q2PH9-1* was detected under both ET treatment and untreated control, contributing 10% to phenotypic variance in PH. QTL *q2PH8-1* was identified only under ET treatment, and it explained over 10% of PH phenotypic variance.

### QTL analysis for ear height

For EH, 17 QTLs in Pop. 1, and 8 in Pop. 2 were identified on all chromosomes except for chromosome 10. An individual QTL explained 4.46 to17.71% of phenotypic variance. Four of the 17 QTLs from KUI3 (*q2EH2-3*, *q2EH5*, *q2EH7*, *q2EH9*) resulted in an increase in trait values. Four QTLs (*q2EH2-3*, *q2EH6*, *q2EH7*, *q2EH9*) were detected under ET-treated and control conditions, with two QTLs (*q2EH6*, *q2EH7*) explaining about 10% of phenotypic variance. Two QTLs (*q2EH2-2*, *q2EH5*) were determined only under control conditions.

### QTL analysis for internode length above the uppermost ear

Eighteen QTLs were detected for ILAU: 10 in Pop. 1, and 8 in Pop. 2. These QTLs were mapped to every chromosome except for chromosomes 4 and 5. The contributions to phenotypic variance for an individual QTL ranged from 3.87 to 13.80%, and with four QTLs contributing about 10%. Five alleles from 18 QTLs were inherited by K22 (*q1ILAU2-2*, *q1ILAU3*, *q1ILAU8*, *q1ILAU10*) and KUI3 (*q2ILAU9*), and led to a rise in trait values. Seven QTLs (*q1ILAU2-2*, *q1ILAU6*, *q1ILAU7-1*, *q1ILAU8*, *q1ILAU9-1*, *q2ILAU2-2*, *q2ILAU9*) were identified in ET-treated and control conditions, while three QTLs (*q1ILAU2-1*, *q1ILAU7-2*, *q2ILAU8-2*) were identified only under control conditions. QTLs *q1ILAU2-2* and *q2ILAU9*, detected under both ET-treated and control conditions, contributed 10% of phenotypic variance. QTL *q2ILAU8-2*, detected only in control conditions, accounted for more than 10% of the phenotypic variance in ILAU.

### Mapping results comparison

By comparing mapping results, one QTL for three measured traits (*q2PH9-1*, *q1PH9*, *q1EH9*/*q1ILAU9-1*, *q2ILAU9* and *q2EH9*) was consistent across two populations, and two QTLs were consistent for one or two traits (*q2PH2-5*, *q2ILAU2-2*, *q1PH2-2* and *q1ILAU2-2*; *q1PH8-1*, *q1EH8-1*, *q2PH8-1*) across two populations at two locations under both ET-treated and control conditions. Moreover, three QTLs for two or all traits were consistent across one population at both locations under both treatment conditions, four QTLs for two traits across one population at both locations under control conditions, and three QTLs for two traits across one or both populations at both locations under ET treatment (Table 5, Figs 1 and 2).

**Table 5:** Comparison of mapping results across the two RIL populations or ethylene treatments in two locations.

QTL	Location ^a^	Treatment ^b^	Marker Interval ^c^	Support interval ^d^ (cM)	Physical interval ^e^ (bp)	Rang of R^2^ (%)	Candidate Gene/Pevious Studies for PH-related traits
*q1ILAU2-1*	WQ, LS	CK	PZE-102027559- PZE-102028305	41.5–41.8	12,801,930–13,300,441	4.92–7.46	AT4G00880,
*q2PH2-1*	WQ, LS	CK, ET	PZE-102038161- SYN29038	47.1–51.7	18,316,951–20,724,795	6.45–14.91	Wang, 2014; AT2G35700
*q2EH2-2*/*q2PH2-2*	WQ, LS	CK	PZE-102054526- SYN34233	66.1–67.5	32,516,815–36,590,474	5.81–11.31	
*q2PH2-3*/*q2EH2-3*	WQ, LS	ET	PZE-102132131-PZE-102139383	119.2–130.1	182,620,244–187,633,354	5.07–9.25	AT1G72360
*q2PH2-5*/*q2ILAU2-2*/*q1PH2-2*/*q1ILAU2-2*	WQ, LS	CK, ET	PZE-102184387- PZE-102193904	178.5–182.7	227,531,180–228,895,610	4.81–11.07	
*q1PH4*/*q1EH4*	WQ, LS	ET	PZE-104148574- PZE-104148574	142.4–147.3	235,704,291–236,882,332	5.54–5.99	
*q2PH5*/*q2EH5*	WQ, LS	CK	PZE-105156919- PZE-105158393	162.3–165.8	205,567,299–206,188,028	6.93–9.98	Nikolic et al. 2011; Tang et al. 2012; Wang, 2014; Yang et al. 2008; Zhang et al. 2010; Ku et al. 2014; Ajmone-Marsan et al. 1994; Lübberstedt et al. 1997; Tang et al. 2007; Gonzalo et al. 2010
*q1PH6-1*/*q1ILAU6*	WQ, LS	CK, ET	PZE-106020696- PZE-106013766	18.3–21.9	16,795,395–34,863,238	5.21–10.99	AT1G74930
*q2EH6*	WQ, LS	CK, ET	SYN23640- PZE-106112056	113.6–117.8	156,879,515–159,464,206	4.47–8.56	AT1G78440
*q1PH7*/*q1EH7*/*q1ILAU7-1*	WQ, LS	CK, ET	PZE-107016972- PZE-107021928	52–57.3	14,462,136–21,509,620	4.48–10.99	
*q1PH7-2*/*q1ILAU7-2*	WQ, LS	CK	PZE-107069594- SYN28758	68.6–70.8	126,402,635–129,967,283	4.69–6.90	Wang, 2014; Yang et al. 2008; Lima et al. 2006
*q2EH7*	WQ, LS	CK, ET	PZE-107074506- PZE-107080831	94.6–98.5	130,360,641–133,374,241	4.46–10.16	AT1G73730
*q1PH8-1*/*q1EH8-1*/*q2PH8-1*	WQ, LS	ET	PZE-108009997- SYN3278	42.1–44.8	10,393,147–13,203,852	5.1–12.15	
*q2PH8-3*/*q2ILAU8-2*	WQ, LS	CK	PZE-108064061- SYNGENTA14910	70.8–72	114,297,738–118,401,344	9.74–14.47	
*q1PH8-2*/*q1ILAU8*	WQ, LS	CK, ET	PZE-108099425- PZE-108105216	90.3–98.3	155,643,006–159,952,552	4.73–9.27	
*q2PH9-1*/*q1PH9*/*q1EH9*/*q1ILAU9-1*/*q2ILAU9*/*q2EH9*	WQ, LS	CK, ET	PZE-109022525- PZE-109027610	46.3–50.6	23,008,509–28,005,182	5.62–13.01	Wang, 2014; Sibov et al. 2003; Yang et al. 2008; Gonzalo et al. 2010; dwarf3

*PH* plant height, *EH* ear height, *ILAU* the internode length above the uppermost ear.

^a^ *Location* QTL detected in Wuqiao (WQ) and Lishu (LS).

^b^ *Treatment CK*, without ethylene (CK) treatments; *ET*, with ethylene (ET) treatments.

^c^ *Marker Interval* Flanking markers, the left and right markers of the QTL.

^d^ *Support interval* Genetic position interval of each QTL.

^e^ *Physical interval* Physical location of the QTL in the maize genome (MaizeDB; http://www.maizegdb.org/).

## Discussion

PH is in highly correlation with biomass yield as it has a large impact on grain yield, shorter plants are more lodging-resistant and have an improved per unit yield [18, 33, 43]. However, even though PH is an important agronomic trait the molecular mechanisms that underlie natural variation remains very elusive in experimental population genetics. A number of studies have shown that hormonally mediated pathways and their interactions are major determinants of PH [44], however, further study of ET-treated PH change is required to uncover the molecular genetic basis of this relationship. Although ethephon application is used in maize to prevent lodging by decreasing PH and EH, the genetic basis of treatments that affect PH is yet unknown. In the present study, PH-related traits with lodging resistance were examined with and without ET treatment using two maize RIL populations. Our results indicate that ET treatment has such a great impact on PH-related traits by decreasing phenotypic performance of PH, EH, and ILAU (*P* < 0.01). Sixty trait-related QTLs were identified under ET-treated and control groups in two populations by CIM, locating 30 QTLs in Pop. 1 and 30 in Pop. 2 (Table 4). Twenty-two QTLs were simultaneously detected in both ET-treated and control conditions, and five QTLs were detected at two geographic locations only under ET treatment. An individual QTL explained 3.87 to 17.71% of the phenotypic variance. One QTL for three measured traits (*q2PH9-1*, *q1PH9*, *q1EH9*/*q1ILAU9-1*, *q2ILAU9* and *q2EH9*) was consistent across both populations, and two QTLs for one or two traits (*q2PH2-5*, *q2ILAU2-2*, *q1PH2-2*, and *q1ILAU2-2*, *q1PH8-1*, *q1EH8-1*, *q2PH8-1*) were identified in both RIL populations at both locations under ET-treated and control conditions. These consistent and stable regions are important QTLs indicating potential hot spots for location of genes of PH, EH, and ILAU responses to ET in maize; therefore, fine-mapping of QTLs and of putative candidate gene validation should enable the cloning of PH, EH, and ILAU related genes to ET response. The data produced in the present study will be of value for further fine-mapping, determination of quantitative trait nucleotides (QTNs), and elucidation of the molecular mechanisms underpinning PH, EH, and ILAU responses to ET.

### Mapping results comparison with previous studies

Many of the QTLs exhibited in this study consistency and stability detected across with differently genetic populations of previously studies in the same locus or adjacent bins (Table 5): alleles for PH in the interval between PZE-102038161 and SYN29038 on chromosome 2 for PH identified across Pop. 1 under both treatment conditions examined here and in a previous study [44]; alleles for PH in the interval between PZE-105156919 and PZE-105158393 on chromosome 5 were identified here in Pop. 2 under control conditions and previous studies [24, 30, 45–51]; between PZE-107069594 and SYN28758 on chromosome 7 for PH and ILAU in Pop. 1 under control conditions in this and previous studies [45, 52]; and alleles for the three measured traits in the interval between PZE-109022525 and PZE-109027610 on chromosome 4 were identified in the two population under both ET-treated and control conditions and in previous studies [30, 45, 48, 53]. Taken together, these findings show that many QTLs influence PH, EH and ILAU in maize, suggesting a common origin among some traits. In addition, three clustered QTLs regions (*q2PH2-5*/*q2ILAU2-2*/*q1PH2-2*/*q1ILAU2-2*, *q1PH7*/*q1EH7*/*q1ILAU7-1*, and *q1PH8-2*/*q1ILAU8*) detected under at both locations and both ET-treated and control conditions was not identified in previous studies, and contributed to more than 10% of phenotypic variance. These novel and stable robust QTLs for PH, EH, and ILAU, further indicated that the genetic structures of the PH-related trait in response to ET treatment was affected by many different minor effective QTLs.

### Comparison of the mapping results across the measured traits in the two RIL populations

We verified three clustered QTLs (*q2PH2-5*/*q2ILAU2-2*/*q1PH2-2*/q1ILAU2-2, *q1PH8-1*/*q1EH8-1*/*q2PH8-1*, and *q2PH9-1*/*q1PH9*/*q1EH9*/*q1ILAU9-1*/*q2ILAU9*/*q2EH9*) located in the same or similar chromosomes regions of the two populations (Pop. 1, and Pop. 2, Table 5), demonstrate that the traits may also be regulated by one QTL or several of the same QTLs. At the same time, the several other QTLs associated with PH-related traits in response to ET treatment were identified among the different populations. Six QTLs (e.g., *q1ILAU2-1*, *q1PH4*/*q1EH4*) were identified for one or more measured trait in Pop. 1, while other seven QTLs (e.g., *q2PH2-1*, *q2EH2-2*/*q2PH2-2*) only detected in Pop. 2. The results demonstrate that PH-related traits in response to ET treatment in maize can be affected by population-specific QTLs, which attributed to differences in the genetic backgrounds of two populations due to the parental line different.

### Genetic architecture of the ethylene response

As a phytohormone, ET has been shown to be involved in stem elongation in deepwater rice [54]. However, some literatures indicated that exogenous ET with moderate to high concentration inhibited stem elongation of maize, as well as other cereal crops such as wheat, oat, and barley etc. [17, 18]. Our results showed that ET treatment applied to maize had similar the physiological responses (i.e. decreasing phenotypic performance of PH, EH, and ILAU) to abiotic stress factors (plant density, waterlogging, etc.). For the three agronomic traits, 19 genetic loci of mapping QTLs were identified as ET-responsive loci in Pop. 1, and 22 genetic loci sensitive to ET in Pop. 2. Further, 7 ET-specific QTLs (11.67%) identified in the two RIL populations. Thus, the maize ET responses assessed here were consistent and specific, as those described in other studies such stressors such as plant density, nitrogen deficiency and waterlogging [34, 55–58]. The results of present study could provide a valuable reference for finding specific genes and elucidating the molecular mechanism involved in ET responses.

### Associations among QTLs and candidate genes in maize

To explore further the molecular mechanism of PH-related trait variance, the association from QTLs to genes known to be found in PH-related traits in *Arabidopsis* (a model dicotyledonous plant), rice (a monocotyledonous plant), and maize were studied by the bioinformatics approach using the *Zea mays* genome (Table 5). Sequences for candidate genes of *Arabidopsis*, and crops e.g. maize and rice were downloaded from the National Center for Biotechnology Information (NCBI), and their homologs in maize inbred line B73 were investigated using maizeGDB blast with an *E*-value cutoff of 10^−10^ and coverage longer than 60% [42]. Seven candidate genes controlling PH-related traits were located in 7 consistent QTL intervals (Table 5). *AT2G35700*, *AT1G72360*, and *AT1G74930* were found to be located in the *q2PH2-1*, *q2PH2-3*/*q2EH2-3*, and *q1PH6-1*/*q1ILAU6* intervals, respectively. The two genes encode a member of the subfamily of ERF/AP2 transcription factors. Indeed, the *AP2* genes belong to a large gene family that encode a highly conserved AP2/ERF DNA binding domain and are importance in the regulation of development and in responses to abiotic and biotic stresses [59–61]. Mutations near the exon-intron boundaries in these genes cause misspliced transcript variants, and result in phenotypic changes to the plants, specifically shorter internodes and wrinkled leaves [61]. *AT4G00880* was located in the *q1ILAU2-1* interval. The gene encodes a small size auxin-induced protein initially identified in *Arabidopsis*, soybean, and later in other plants [62–65]. A few SAUR proteins are shown to bind CaM [61], alter apical hook development, and negatively regulate auxin synthesis and transport [64, 66]. Proteins *SAUR76*, *77*, and *78* integrate auxin into ethylene signaling to regulate ET response and plant growth [64]. *AT1G78440* was located in the *q2EH6* interval. The gene encodes a gibberellin 2-oxidase (*GA2ox*) that acts on C19 gibberellins. *GA2ox* hydrolyzes carbon-2 positions of bioactive gibberellic acids (GA1, GA4) and immediate precursors (GA9, GA12, GA20, and GA53) to inactive these proteins [67]. In *Arabidopsis thaliana* and *Nicotiana tabacum*, overexpression of *AtGA2ox7* or *AtGA2ox8* has been shown to result in decreased levels of active gibberellic acids (GAs) and to induce extremely dwarf phenotypes [68]. Moreover, transgenic *Torenia fournieri* plants overexpressing *TfGA2ox* showed dwarf phenotypes as well. However, a mutant of the *SLENDER* gene of *Pisum sativum* encoding *GA2ox*, had higher PH and accumulated GA precursors of high concentration in seeds [69]. Therefore, manipulation of gibberellic acid metabolism by *GA2ox* overexpression might be effective to modify PH. *AT1G73730* was located in the *q1PH7-2*/*q1ILAU7-2* interval. This gene encodes ethylene insensitive 3-like protein (*EIN3*), a transcription factor involving ET signal transduction pathway in *Arabidopsis* [70]. *EIN3* and various *EIN3-like* proteins are not ET-induced but are regulated at post-transcriptional level. Transcription factors can act as activators or repressors of additional downstream ET-responsive genes. Transgenic rice plants overexpressing *OsEIL1* (an *EIN3-like* gene) have been shown to exhibit a short shoot phenotype, coiled primary root, short root, and elevated response to exogenous ET [3]. *Dwarf3* was located in the *q2PH9-1*/*q1PH9*/*q1EH9*/*q1ILAU9-1*/*q2ILAU9*/*q2EH9* interval. This gene encodes a cytochrome P450-mediated early step in gibberellin biosynthesis in maize [61]. Allelic variation at the *Dwarf 3* locus is proposed as basis of a QTL, which was defined for a natural maize height variant [71, 72]. The functional analysis of homologous genes confirm that the maize ET responses QTLs involves ET signal transduction cascade and also interacting with other plant hormones.

The QTLs and candidate genes identified will be of great value for fine-mapping and quantitative nucleotide determination to QTLs cloning of ET-responsive PH-related traits in maize [29, 73–74]. Therefore, further deep understanding for the mapping alleles may contribute to enhancing efficiency for plant height-related traits genetic improvement and elucidating the molecular mechanism involved in ET responses.

## Supporting information

S1 Table Phenotypic data in Pop. 1 for the study. (XLSX)

S2 Table Phenotypic data in Pop. 2 for the study. (XLSX)

## References

[1] Yang SF, Hoffman NE (1984) Ethylene biosynthesis and its regulation in higher plants. Annual review of plant physiology 35: 155–189.

[2] Wang Q, Zhang W, Yin Z, Wen CK (2013) Rice CONSTITUTIVE TRIPLE-RESPONSE2 is involved in the ethylene-receptor signalling and regulation of various aspects of rice growth and development. J Exp Bot 64: 4863–4875. doi: 10.1093/jxb/ert272 24006427 10.1093/jxb/ert272 PMC3830475

[3] Mao C, Wang S, Jia Q, Wu P (2006) OsEIL1, a rice homolog of the Arabidopsis EIN3 regulates the ethylene response as a positive component. Plant molecular biology 61: 141–152. doi: 10.1007/s11103-005-6184-1 16786297 10.1007/s11103-005-6184-1

[4] Qi W, Sun F, Wang Q, Chen M, Huang Y, Feng Y, et al (2011) Rice ethylene-response AP2/ERF factor OsEATB restricts internode elongation by down-regulating a gibberellin biosynthetic gene. Plant Physiol 157: 216–228. doi: 10.1104/pp.111.179945 21753115 10.1104/pp.111.179945 PMC3165871

[5] Li J, Sima W, Ouyang B, Wang T, Ziaf K, Luo Z, et al (2012) Tomato SlDREB gene restricts leaf expansion and internode elongation by downregulating key genes for gibberellin biosynthesis. Journal of experimental botany 63: 6407–6420. doi: 10.1093/jxb/ers295 23077200 10.1093/jxb/ers295 PMC3504492

[6] Abeles FB, Morgan PW, Saltveit ME Jr (2012) Ethylene in plant biology Academic press.

[7] Ma B, Chen S, Zhang J (2010) Ethylene signaling in rice. Chinese Science Bulletin 55: 2204–2210.

[8] Schaller GE (2012) Ethylene and the regulation of plant development. BMC biology 10: 9 doi: 10.1186/1741-7007-10-9 22348804 10.1186/1741-7007-10-9 PMC3282650

[9] Stepanova AN, Alonso JM (2009) Ethylene signaling and response: where different regulatory modules meet. Curr Opin Plant Biol 12: 548–555. doi: 10.1016/j.pbi.2009.07.009 19709924 10.1016/j.pbi.2009.07.009

[10] Merchante C, Alonso JM, Stepanova AN (2013) Ethylene signaling: simple ligand, complex regulation. Curr Opin Plant Biol 16: 554–560. doi: 10.1016/j.pbi.2013.08.001 24012247 10.1016/j.pbi.2013.08.001

[11] Kieber JJ, Ecker JR (1993) Ethylene gas: it's not just for ripening any more! Trends in Genetics 9: 356–362. 8273151 10.1016/0168-9525(93)90041-f

[12] Dugardeyn J, Van Der Straeten D (2008) Ethylene: Inhibitor and stimulator of plant growth Plant Growth Signaling: Springer pp. 199–221.

[13] Cohen E, Kende H (1987) In vivo 1-aminocyclopropane-1-carboxylate synthase activity in internodes of deepwater rice enhancement by submergence and low oxygen levels. Plant Physiology 84: 282–286. 16665431 10.1104/pp.84.2.282 PMC1056571

[14] Fukao T, Bailey-Serres J (2008) Ethylene-A key regulator of submergence responses in rice. Plant Science 175: 43–51.

[15] Hoffmann-Benning S, Kende H (1992) On the role of abscisic acid and gibberellin in the regulation of growth in rice. Plant Physiology 99: 1156–1161. 16668983 10.1104/pp.99.3.1156 PMC1080597

[16] Hattori Y, Nagai K, Furukawa S, Song XJ, Kawano R, Sakakibara H, et al (2009) The ethylene response factors SNORKEL1 and SNORKEL2 allow rice to adapt to deep water. Nature 460: 1026–1030. doi: 10.1038/nature08258 19693083 10.1038/nature08258

[17] Rajala A, Peltonen-Sainio P, Onnela M, Jackson M (2002) Effects of applying stem-shortening plant growth regulators to leaves on root elongation by seedlings of wheat, oat and barley: mediation by ethylene. Plant Growth Regulation 38: 51–59.

[18] Shekoofa A, Emam Y (2008) Plant growth regulator (ethephon) alters maize (Zea mays L.) growth, water use and grain yield under water stress. Journal of Agronomy 7: 41.

[19] Norberg O, Mason S, Lowry S (1988) Ethephon influence on harvestable yield, grain quality, and lodging of corn. Agronomy journal 80: 768–772.

[20] Clark R, Fedak G (1977) Effects of chlormequat on plant height, disease development and chemical constituents of cultivars of barley, oats, and wheat. Canadian Journal of Plant Science 57: 31–36.

[21] Rajala A (2004) Plant growth regulators to manipulate oat stands. Agricultural And Food Science 13: 186–197.

[22] Rajala A, Peltonen-Sainio P (2008) Timing applications of growth regulators to alter spring cereal development at high latitudes. Agricultural and Food Science 11: 233–244.

[23] Weyers JD, Paterson NW (2001) Plant hormones and the control of physiological processes. New Phytologist 152: 375–407. 10.1046/j.0028-646X.2001.00281.x 33862994

[24] Ku L, Zhang L, Tian Z, Guo S, Su H, Ren Z, et al (2015) Dissection of the genetic architecture underlying the plant density response by mapping plant height-related traits in maize (Zea mays L.). Molecular Genetics and Genomics: 1–11. 10.1007/s00438-014-0987-1 25566854

[25] Lv H, Zheng J, Wang T, Fu J, Huai J, Min H, et al (2014) The maize d2003, a novel allele of VP8, is required for maize internode elongation. Plant Mol Biol 84: 243–257. doi: 10.1007/s11103-013-0129-x 24214124 10.1007/s11103-013-0129-x

[26] Teng F, Zhai L, Liu R, Bai W, Wang L, Huo D, et al (2013) ZmGA3ox2, a candidate gene for a major QTL, qPH3.1, for plant height in maize. Plant J 73: 405–416. doi: 10.1111/tpj.12038 23020630 10.1111/tpj.12038

[27] Peiffer JA, Flint-Garcia SA, De Leon N, McMullen MD, Kaeppler SM, Buckler E. (2013) The genetic architecture of maize stalk strength. PLoS One 8: e67066 doi: 10.1371/journal.pone.0067066 23840585 10.1371/journal.pone.0067066 PMC3688621

[28] Bai W, Zhang H, Zhang Z, Teng F, Wang L, Tao Y, et al (2009) The evidence for non-additive effect as the main genetic component of plant height and ear height in maize using introgression line populations. Plant Breeding.

[29] Zhang W, Zhang M, Li Z, Duan L. (2017) Dissection of the molecular genetic architecture of the ratio of ear to plant heights in response to ethylene by a RIL population with SNPs marker in maize. Acta Physiologiae Plantarum 39: 142.

[30] Gonzalo M, Holland J, Vyn T, McIntyre L (2010) Direct mapping of density response in a population of B73× Mo17 recombinant inbred lines of maize (Zea mays L.). Heredity 104: 583–599. doi: 10.1038/hdy.2009.140 19888291 10.1038/hdy.2009.140

[31] Wei X (2011) Study on Regulation Mechanism of ethephon on Internode Elongation in Maize. PhD Dissertation of China Agricultural University: 14–19.

[32] Abendroth LJ, Elmore R, Boyer M, Marlay S (2011) Corn growth and development: Iowa State University, University Extension.

[33] Ye DL, Zhang YS, Al-Kaisi MM, Duan LS, Zhang MC, and Li ZH (2015) Ethephon improved stalk strength associated with summer maize adaptations to environments differing in nitrogen availability in the North China Plain. The Journal of Agricultural Science: 1–18.

[34] Ku L, Ren Z, Chen X, Shi Y, Qi J, Su H, et al (2016) Genetic analysis of leaf morphology underlying the plant density response by QTL mapping in maize (Zea mays L.). Molecular Breeding 36: 1–16.

[35] Raihan MS, Liu J, Huang J, Guo H, Pan Q, and Yan, J (2016) Multi-environment QTL analysis of grain morphology traits and fine mapping of a kernel-width QTL in Zheng58× SK maize population. Theoretical and Applied Genetics: 1–13. 10.1007/s00122-016-2717-z 27154588

[36] Andersen JR, Schrag T, Melchinger AE, Zein I, Lübberstedt T (2005) Validation of Dwarf8 polymorphisms associated with flowering time in elite European inbred lines of maize (Zea mays L.). Theoretical and Applied Genetics 111: 206–217. doi: 10.1007/s00122-005-1996-6 15933874 10.1007/s00122-005-1996-6

[37] Saghai-Maroof MA, Soliman KM, Jorgensen RA, Allard R (1984) Ribosomal DNA spacer-length polymorphisms in barley: Mendelian inheritance, chromosomal location, and population dynamics. Proceedings of the National Academy of Sciences 81: 8014–8018. 10.1073/pnas.81.24.8014 PMC392284 6096873

[38] Pan Q, Li L, Yang X, Tong H, Xu S, Li Z, et al (2015) Genome-wide recombination dynamics are associated with phenotypic variation in maize. New Phytol. 10.1111/nph.13810 26720856

[39] Chen G, Wang X, Long S, Jaqueth J, Li B, Yan J, et al (2016) Mapping of QTL conferring resistance to northern corn leaf blight using high-density SNPs in maize. Molecular Breeding 36: 1–9.

[40] Wang S, Basten C, Zeng Z (2007) Windows QTL Cartographer 2.5. Department of Statistics, North Carolina State Univ., Raleigh, NC.

[41] Han Z, Ku L, Zhang Z, Zhang J, Guo S, Liu H, et al (2014) QTLs for seed vigor-related traits identified in maize seeds germinated under artificial aging conditions. PloS one 9: e92535 doi: 10.1371/journal.pone.0092535 24651614 10.1371/journal.pone.0092535 PMC3961396

[42] Guo S, Ku L, Qi J, Tian Z, Han T, Zhang L, et al (2015) Genetic Analysis and Major Quantitative Trait Locus Mapping of Leaf Widths at Different Positions in Multiple Populations. PloS one 10: e0119095 doi: 10.1371/journal.pone.0119095 25756495 10.1371/journal.pone.0119095 PMC4354904

[43] Zhang Q, Zhang L, Evers J, van der Werf W, Zhang W, and Duan L (2014) Maize yield and quality in response to plant density and application of a novel plant growth regulator. Field Crops Research 164: 82–89.

[44] Wang Y, Li J (2008) Molecular basis of plant architecture. Annu Rev Plant Biol 59: 253–279. doi: 10.1146/annurev.arplant.59.032607.092902 18444901 10.1146/annurev.arplant.59.032607.092902

[45] Wang B (2014) Combining Large Recombinant Inbred Lines Population and Ultra-high Density Molecular Markers to Identify QTL for Important Agronomic Traits in Maize. PhD Dissertation of China Agricultural University.

[46] Tang J, Teng W, Yan J, Ma X, Meng Y, Dai J, et al (2007) Genetic dissection of plant height by molecular markers using a population of recombinant inbred lines in maize. Euphytica 155: 117–124.

[47] Lübberstedt T, Melchinger AE, Schön CC, Utz HF, Klein D (1997) QTL mapping in testcrosses of European flint lines of maize: I. Comparison of different testers for forage yield traits. Crop science 37: 921–931.

[48] Yang X, Lu M, Zhang S, Zhou F, Qu Y, Xie C. (2008) QTL mapping of plant height and ear position in maize (Zea mays L.). Hereditas 30: 1477–1486. 19073558 10.3724/sp.j.1005.2008.01477

[49] Zhang Y, Li Y, Yang W, Liu Z, Cheng L, Bo P, et al (2010) Stability of QTL across environments and QTL-by-environment interactions for plant and ear height in maize. Agricultural Sciences in China 9: 1400–1412.

[50] Nikolić S, Mojović L, Rakin M, Pejin D, Pejin J (2011) Utilization of microwave and ultrasound pretreatments in the production of bioethanol from corn. Clean Technologies and Environmental Policy 13: 587–594.

[51] Tang Z, Yang Z, Hu Z, Zhang D, Lu X, Jia B, et al (2013) Cytonuclear epistatic quantitative trait locus mapping for plant height and ear height in maize. Molecular breeding 31: 1–14.

[52] Lima MdLA, de Souza CL Jr, Bento DAV, de Souza AP, Carlini-Garcia LA (2006) Mapping QTL for grain yield and plant traits in a tropical maize population. Molecular Breeding 17: 227–239.

[53] Sibov ST, de Souza J, Lopes C, Garcia A.A, Silva AR, Garcia A et al (2003) Molecular mapping in tropical maize (Zea mays L.) using microsatellite markers. 2. Quantitative trait loci (QTL) for grain yield, plant heigth, ear height and grain moisture. Hereditas 139: 107–115. doi: 10.1111/j.1601-5223.2003.01667.x 15061811 10.1111/j.1601-5223.2003.01667.x

[54] Van Der Straeten D, Zhou Z, Prinsen E, Van Onckelen HA, Van Montagu MC (2001) A comparative molecular-physiological study of submergence response in lowland and deepwater rice. Plant Physiology 125: 955–968. 11161052 10.1104/pp.125.2.955 PMC64896

[55] Sari-Gorla M, Krajewski P, Di Fonzo N, Villa M, Frova C (1999) Genetic analysis of drought tolerance in maize by molecular markers. II. Plant height and flowering. Theoretical and Applied Genetics 99: 289–295.

[56] Messmer R, Fracheboud Y, Banziger M, Vargas M, Stamp P, and Ribaut J (2009) Drought stress and tropical maize: QTL-by-environment interactions and stability of QTLs across environments for yield components and secondary traits. Theor Appl Genet 119: 913–930. doi: 10.1007/s00122-009-1099-x 19597726 10.1007/s00122-009-1099-x

[57] Osman KA, Tang B, Wang Y, Chen J, Yu F, Li L, et al (2013) Dynamic QTL analysis and candidate gene mapping for waterlogging tolerance at maize seedling stage. PloS one 8: e79305 doi: 10.1371/journal.pone.0079305 24244474 10.1371/journal.pone.0079305 PMC3828346

[58] Zhang X, Tang B, Yu F, Li L, Wang M, Xue Y, et al (2013) Identification of Major QTL for Waterlogging Tolerance Using Genome-Wide Association and Linkage Mapping of Maize Seedlings. Plant Molecular Biology Reporter 31: 594–606.

[59] Riechmann JL, Meyerowitz EM (1998) The AP2/EREBP family of plant transcription factors. Biological chemistry 379: 633–646. 9687012 10.1515/bchm.1998.379.6.633

[60] Zhuang J, Deng D, Yao Q, Zhang J, Xiong F, Chen J, et al (2010) Discovery, phylogeny and expression patterns of AP2-like genes in maize. Plant Growth Regulation 62: 51–58.

[61] Jiang F, Guo M, Yang F, Duncan K, Jackson D, et al (2012) Mutations in an AP2 Transcription Factor-Like Gene Affect Internode Length and Leaf Shape in Maize. PLoS ONE 7: e37040 doi: 10.1371/journal.pone.0037040 22649507 10.1371/journal.pone.0037040 PMC3359370

[62] Yang T, Poovaiah B (2000) Molecular and biochemical evidence for the involvement of calcium/calmodulin in auxin action. Journal of Biological Chemistry 275: 3137–3143. 10652297 10.1074/jbc.275.5.3137

[63] Hagen G, Guilfoyle T (2002) Auxin-responsive gene expression: genes, promoters and regulatory factors. Plant molecular biology 49: 373–385. 12036261

[64] Kant S, Bi Y-M, Zhu T, Rothstein SJ (2009) SAUR39, a small auxin-up RNA gene, acts as a negative regulator of auxin synthesis and transport in rice. Plant physiology 151: 691–701. doi: 10.1104/pp.109.143875 19700562 10.1104/pp.109.143875 PMC2754634

[65] Li Z, Chen H, Li Q, Tao J, Bian X, Ma B, et al (2015) Three SAUR proteins SAUR76, SAUR77 and SAUR78 promote plant growth in Arabidopsis. Scientific reports 5. 10.1038/srep12477 PMC4513569 26207341

[66] Park JE, Kim YS, Yoon HK, Park CM (2007) Functional characterization of a small auxin-up RNA gene in apical hook development in Arabidopsis. Plant science 172: 150–157.

[67] Lee DH, Lee IC, Kim KJ, Kim DS, Na HJ, Lee IJ, et al (2014) Expression of gibberellin 2-oxidase 4 from Arabidopsis under the control of a senescence-associated promoter results in a dominant semi-dwarf plant with normal flowering. Journal of Plant Biology 57: 106–116.

[68] Schomburg FM, Bizzell CM, Lee DJ, Zeevaart JA, Amasino RM (2003) Overexpression of a novel class of gibberellin 2-oxidases decreases gibberellin levels and creates dwarf plants. The Plant Cell 15: 151–163. doi: 10.1105/tpc.005975 12509528 10.1105/tpc.005975 PMC143488

[69] Martin DN, Proebsting WM, Hedden P (1999) The SLENDER gene of pea encodes a gibberellin 2-oxidase. Plant Physiology 121: 775–781. 10557225 10.1104/pp.121.3.775 PMC59439

[70] Chao Q, Rothenberg M, Solano R, Roman G, Terzaghi W, and Ecker J (1997) Activation of the ethylene gas response pathway in Arabidopsis by the nuclear protein ETHYLENE-INSENSITIVE3 and related proteins. Cell 89: 1133–1144. 9215635 10.1016/s0092-8674(00)80300-1

[71] Prioul JL, Pelleschi S, Séne M, Thévenot C, Causse M, de Vienne D, et al (1999) From QTLs for enzyme activity to candidate genes in maize. Journal of Experimental Botany 50: 1281–1288.

[72] Touzet P, Winkler R, Helentjaris T (1995) Combined genetic and physiological analysis of a locus contributing to quantitative variation. Theoretical and Applied Genetics 91: 200–205. doi: 10.1007/BF00220878 24169764 10.1007/BF00220878

[73] Yang Q., Zhang D., and Xu M. (2012). A sequential quantitative trait locus fine-mapping strategy using recombinant-derived progeny. Journal of integrative plant biology 54, 228–237. doi: 10.1111/j.1744-7909.2012.01108.x 22348858 10.1111/j.1744-7909.2012.01108.x

[74] Xu ML, Li BL, Fengler K, Chao Q, Chen YS, Zhao XR, et al. (2014) Genetic loci associated with head smut resistance in maize. Patent Application No. 14/546,401.

